# Calprotectin Is Associated with HETE and HODE Acids in Inflammatory Bowel Diseases

**DOI:** 10.3390/jcm12247584

**Published:** 2023-12-08

**Authors:** Małgorzata Szczuko, Paulina Komisarska, Justyna Kikut, Arleta Drozd, Diana Sochaczewska

**Affiliations:** 1 Department of Human Nutrition and Metabolomics, Pomeranian Medical University in Szczecin, 72-009 Police, Polandarleta.drozd@pum.edu.pl (A.D.); 2 Department of Neonatology, Pomeranian Medical University in Szczecin, 72-009 Police, Poland; diana.sochaczewska@pum.edu.pl

**Keywords:** Crohn’s disease, ulcerative colitis, calprotectin, arachidonic acid, hydroxyeicosatrienoic acids, hydroxyoctadecadienoic acids, children

## Abstract

Background: Intestinal diseases are identified as autoimmune phenomena attributed to a specific virus that binds to the mucosal epithelium. The importance of precise diagnostic processes and identification is emphasized, but the multifaceted and complex etiological factors pose challenges for effective treatment. A recent supplementary study suggested a linkage between the secretion of calprotectin, a protein associated with inflammatory processes, and increased levels of hydroxyeicosatrienoic acids (HETE) and hydroxyoctadecadienoic (HODE) compounds. Methods: Sixty-two patients (average age: 14.06 ± 2.93 years) suffering from inflammatory bowel diseases were included in this study. Comparative analyses were performed to assess the concentrations of calprotectin against the levels of arachidonic acid derivatives. The calprotectin concentration was determined using the enzyme-linked immunosorbent assay (ELISA) method. The derivatives of HETE and HODE were identified through liquid chromatography. Results: Patients with Crohn’s disease (CD) displayed higher average concentrations of fatty acid metabolites; however, no correlation with calprotectin was observed. A dependency of 12S HETE concentration relative to age was noted in the CD group, and a similar trend was also identified in ulcerative colitis (UC), with the significant metabolites being 15 HETE and 5 oxoETE. In UC patients, a positive correlation was established between the calprotectin concentration and the acids 5-HETE and 12-HETE. Conclusions: These findings may be instrumental for monitoring the inflammatory states of patients and indicating a pathway for intervention. The metabolite 16RS HETE is associated with UC activity, and 15-HETE is related to the disease’s duration. A relatively more significant role of HETE acids in the progression of the disease was observed in UC.

## 1. Introduction

Crohn’s disease (CD) and ulcerative colitis (UC) are classified under the group of inflammatory bowel diseases (IBDs). These conditions are predominantly present in developed countries, especially North America and Northern Europe [1]. In Europe alone, an estimated 3 million people are affected [2], predominantly those aged between 15 and 30, leading to these conditions being termed as diseases of the young [3]. The results of this study suggest a relationship between the secretion of calprotectin, which is associated with inflammatory processes and increased concentrations of hydroxyepoxyeicosatrienoic (HETE) and hydroxyoctadecadieonic (HODE) acids.

### 1.1. Etiopathogenic Factors

The development of these diseases is multifactorial, involving immunological, environmental, and genetic factors. They are characterized by the dysregulation of T lymphocytes, but this is not the only cause of the disease. CD is marked by the overactivity of Th1 lymphocytes, which are responsible for the production of proinflammatory cytokines, while UC is associated with Th2 lymphocytes [4]. Both diseases exhibit neutrophil infiltration in intestinal tissues. In CD, the presence of lymphocytes and their penetration beyond the intestinal mucosal border is notable, whereas in UC, only neutrophils are present, and they do not cross this border [5,6]. Activated neutrophils release acute-phase proteins (e.g., calprotectin) with antibacterial properties, which are utilized in differentiating IBDs from irritable bowel syndrome (IBS) [7]. A higher incidence is observed among first-degree relatives (10–30%) and the Caucasian population, where the likelihood is quadrupled. The diseases’ association with the presence of HLA DR2 and DR5 genome is indicated among the genetic factors [8]. 

Environmental pollutants; smoking; urban living; and a Western diet characterized by high amounts of simple carbohydrates, low amounts of fiber, and high amounts of fat, including saturated fatty acids, omega-6, and trans fats, are known risk factors. Additives in food can combine with bacterial cell wall lipopolysaccharides, forming antigenic complexes that drive inflammatory reactions [9].

### 1.2. IBD Diagnosis and Useful Markers

The primary diagnostic method is endoscopy, coupled with histopathological examinations of intestinal fragments [10,11]. It is invasive, expensive, time-consuming, and requires patient preparation. Although time-consuming and invasive, colonoscopies with biopsies and histopathological exams are standard for establishing an IBD diagnosis; therefore, new methods are being sought. Other markers, like C-reactive protein (CRP), fibrinogen, and erythrocyte sedimentation rate (ESR), are used, but they are less specific [12]. CRP and fibrinogen are useful but non-specific, while calprotectin is more useful since it reflects intestinal inflammation. It has been proven that circulating calprotectin induces the production of TNF-α and interleukins IL-6, IL-1β, and IL-8 in monocytes, playing an important role in inducing and maintaining inflammation. Furthermore, it correlates with disease activity and laboratory inflammatory variables. Calprotectin, a calcium and zinc-binding protein complex, is the most precise marker and is primarily found in neutrophils. It is released during inflammation and is stable against proteolytic degradation due to its calcium-binding capability [13]. Neutrophils secrete calprotectin and cause its increased concentration in feces, limiting the adhesion and growth of pathogenic bacteria to intestinal mucosa cells [13]. It also has chemokine-like properties, stimulating neutrophils to synthesize inteleukin 8 (IL-8) and inducing apoptosis processes. Calprotectin modulates changes in the structure of the cytoskeleton in tissues, enabling the migration of leukocytes and arachidonic acid to the site of inflammation [14,15]. Arachidonic acid undergoes further transformations until its derivatives, HETE and HODE, are formed [14].

### 1.3. Proinflammatory Mediators of Arachidonic Acid

Arachidonic acid (AA) is a precursor to biologically active compounds like leukotrienes, prostaglandins, and thromboxanes, playing roles in inflammation, smooth muscle, and blood platelet aggregation [16]. In the context of IBDs, AA induces chemokine production from intestinal epithelial cells and causes intestinal inflammation in genetically susceptible mice [16,17]. Its release can be induced by neurotransmitters, toxins, among others, and is further metabolized to HETE and HODE derivatives [17,18]. With the participation of cyclooxygenase (COX), prostaglandins and HODE acids are produced [19]. Lipoxygenases stimulate the conversion of AA into 5-hydropyroxyeicosatetraenoic acid (5-HPETE). 5-HPETE is further converted into 5-hydroxyeicosatetraenoic acid (5-HETE). 5-HETE acid is then converted into leukotrienes (LT). LTB4 has a chemotactic effect; it has the strongest effect on neutrophils, and it stimulates the release of lysosomal enzymes and superoxide radicals by neutrophils [17,18,19].

In CD exacerbation, a greater role for the 15-lipoxygenase and 5-lipoxygenase pathways is observed. Patients in remission show higher concentrations of PGE2 and 12-HETE. UC is marked by a deficiency in lipoxins due to the disrupted activity of the 5-LOX and 15-LOX enzymes [11,20]. Eicosanoids made from arachidonic acid are highly biologically active, and even in very small amounts, they can influence the occurrence of strong inflammatory reactions [21].

The aim of this study was to ascertain the relationship between an increased calprotectin content and increased concentrations of HETE and HODE fatty acid metabolite derivatives in inflammatory bowel diseases and the participation of these derivatives in the active phase of the disease, which may be the goal of therapy.

## 2. Materials and Methods 

### 2.1. Characteristics of the Study Group

The study encompassed 62 individuals under 18 years of age suffering from IBDs, including 29 patients with UC and 33 with CD. They were hospitalized in two clinical centers in Poland. The exclusion criteria included those on specialized elimination diets and enteral nutrition regimes. The IBD diagnosis was established based on clinical and imaging criteria (i.e., endoscopic and histopathological examinations). The study group’s characteristics are detailed in Table 1.

The patient group comprised individuals suffering from unspecified inflammatory bowel diseases, i.e., CD and UC. The subjects, who were children and adolescents, had an average age of 14.04 ± 2.93 of age and were mostly in the exacerbation phase of the disease, with a low average BMI and high calprotectin concentration. Both groups had similar primary disease onset times, averaging 11.8 ± 3.93 years. UC patients were characterized by higher average disease activity indices, body weight, height, age, and calprotectin concentrations. Our statistical analysis revealed significant differences in disease activity phases relative to the disease unit. The UC patients exhibited significantly higher disease activity than the CD patients. The treatment methods in both the CD and UC groups were comparable (aminosalicylates 22 and 24; corticosteroids 9 and 11; immunomodulatory drugs 15 and 3; biological medicines 9 and 5, respectively).

### 2.2. Isolation of Calprotectin

Fecal calprotectin (cat no.K6927, Immunodiagnostik AG, Bensheim, Germany) contents were determined using an immunoenzymatic test with human calprotectin reagents involving two antibodies: one biotinylated against calprotectin and the other against the human calprotectin heterodimer. The reference value for calprotectin was <50 μg/g, indicating the absence of intestinal inflammation. A mildly positive result was deemed to range from 50 to 150 μg/g, warranting observation and potential retesting after 6–8 weeks. A result of >150 μg/g signified active intestinal inflammation, necessitating further diagnostics (e.g., a colonoscopy) [22].

### 2.3. Isolation of HETE and HODE

The isolation method enabled the purification and separation of chemical compounds from the samples. HETE and HODE derivatives were extracted from plasma. Blood was drawn from fasting patients and treated with ethylenediaminetetraacetic acid. The conditioned samples were stored at −80 °C until a biochemical analysis of the mediators was conducted. The compounds were then eluted and subjected to liquid chromatography (Agilent Technologies 1260, Cheadle, UK) by using solid-phase extraction RP-18 SPE columns (Agilent Technologies, Cheadle, UK). Each peak’s absorbance spectrum was analyzed quantitatively based on the internal standard calibration in the peaks’ areas. A DAD detector was used to monitor peaks by adsorption at 235 nm for 9-HODE, 13-HODE, 5-HETE, 12-HETE, and 15-HETE; at 280 nm for PGE2 (Prostaglandin B_2_, internal standard) and 5oxoETE, Leukotriene B4, and TXB2; at 210 nm for Prostaglandin E2, 16-HETE; and at 302 nm for 5(S),6(R)-Lipoxin A4, 5(S),6(R), 15(R)-Lipoxin A4. The absorbance spectra of the peaks were analyzed to confirm the identification of analytes [23].

### 2.4. Statistical Analysis

The results were statistically processed using Statistica 13.3 (Statsoft, Krakow, Poland). All parameters’ distributions were checked, with age deviating from normal distribution across the entire study group, necessitating the use of the non-parametric Mann–Whitney test. For normally distributed parameters, an ANOVA was applied. Following the separation of the group into two disease units, the disease activity index deviated from normal distribution, prompting the use of the Spearman test. Correlation analysis between calprotectin and disease activity with lipid mediators was also performed. *p* < 0.05 was considered statistically significant.

## 3. Results

### 3.1. Comparison of Both Disease Units

Table 2 compares the average arachidonic acid derivatives, considering the disease units. CD was mostly characterized by higher concentrations of fatty acid derivatives, but they were not statistically significantly different (Table 2).

In Table 3, the concentrations of HETE and HODE fatty acid derivatives in the IBD cases are compared, considering the disease unit and disease activity. A correlation was found between ulcerative colitis and the disease activity, and the concentration of 16RS HETE was higher. In CD, as the disease activity decreased, the concentration of 5oxo ETE increased (Table 3). When analyzing the correlations of calprotectin in the overall IBD group and considering the disease units (CD and UC), no statistically significant correlations were found (Table 4). After separating both disease units, it was found that in UC, significant correlations occurred in relation to 12-HETE and 5-HETE, with an increase in the concentration of KT derivatives relative to the increasing level of calprotectin. In CD, the level of calprotectin was not related to the AA derivatives. Regarding disease activity in both disease units (CD and UC), again, no significant dependencies were found. However, in UC, there was a correlation with the concentration of 16RS HETE, while in CD, a high negative correlation with 5 oxoETE was observed (Table 4).

### 3.2. Summary of Research Findings

1. Both disease (CD and UC) units should be considered separately, as when they are considered as a whole group (IBD group), they do not show any correlation with respect to the level of lipid derivatives. However, differences are visible when they are analyzed separately.
2. The CD patients were characterized by slightly higher concentrations of proinflammatory fatty acid metabolites on average, suggesting a more severe course of CD compared to UC involving lipid mediators.
3. Calprotectin concentrations were positively correlated with 12-HETE and 5-HETE within UC only. Such dependencies were not observed in CD.
4. Disease activity in UC was associated with the activation of the 16 RS HETE pathway.
5. Disease activity in CD was negatively associated with the 5-oxo-ETE pathway.

## 4. Discussion

Inflammatory bowel diseases (IBDs) are classified among diseases with high prevalence and an increasing incidence rate. However, they are known to arise from multifactorial reactions [24]. Genetic factors play a significant role, and research also indicates the impact of abnormal gut microbiota and the involvement of Toll-like receptor 4 (TLR4) [25,26]. An abnormal reaction occurs in cells located in the gut-associated lymphoid tissue (GALT) [27]. The characteristics of intestinal lymphoid tissue are dependent on the macrophages, lymphocytes, granulocytes, and cytokines secreted by the cells present within it. The lymphoid system is vital to the interaction process between tolerance to antigens entering the intestines with food and protecting our bodies against pathogens such as fungi, bacteria, and viruses. GALT supports the maintenance of intestinal microbiological homeostasis. During the secretion of inflammatory cytokines by cells in the GALT, tight junctions become leaky, resulting in increased intestinal permeability [28,29]. This damage to the intestinal mucosa impairs the absorption of nutrients and food components and allows harmful substances to enter the circulatory system from the intestinal lumen [30,31]. Making a definitive diagnosis and monitoring disease activity using current methods is not easy, and these tasks usually take a significant amount of time [32]. Despite various therapeutic treatments (biological treatment, steroid therapy, and immunosuppression), some patients do not respond positively to treatment or become immune to the drugs used in their treatment [32,33,34]. 

HETE and HODE acids are metabolites of arachidonic acid released under the influence of phospholipase in inflammatory states involving the intestinal mucosa [34]. In one study, a higher concentration of 12-HETE was observed, along with an elevated level of calprotectin. It has been proven that 12-HETE exerts a chemotactic action on neutrophils [35]. Studies have shown a greater involvement of the 12-LOX pathway in ulcerative colitis (UC) compared to Crohn’s disease (CD) [36]. 5-HETE, like 12-HETE, has higher activity in UC. High concentrations of 5-HETE during flare ups have previously been identified in studies on IBDs, particularly in studies on UC [37]. In the aforementioned studies, the concentrations of these metabolites were positively correlated with the levels of calprotectin. Studies have also shown a higher activity of 5-LOX in patients compared to the healthy control group. 5-HETE is a strong activator of neutrophilia [8,38]. These results are consistent with a study conducted by Masoodi and others. They used a biopsy of the mucous membrane, where increased concentrations of PGE2, PGD2, TXB2, and 5-,11-,12-,15-HETE were found compared to a control group consisting of healthy individuals. Results can vary due to the different tested materials taken from patients [39]. In the conducted study, the CD patient group did not show any significant correlation associated with the arachidonic acid derivatives HETE and HODE or calprotectin concentration. This dependency may be related to the lack of correlation with increased calprotectin concentration and the flare-up phase in CD; not every patient with an IBD has this dependency [27]. However, a correlation was observed in the case of the disease activity index. Scientific studies have also suggested the greater involvement of HODE acids compared to HETE acids in CD [8].

In summary, based on the results obtained in this work and studies published by other authors, it can be concluded that not all arachidonic acid derivatives acted in the same way on the entire group of patients with an IBD. They differentiate depending on the disease unit, duration, and activity of the disease. An essential point of the above studies is the correlation of elevated concentrations of 5-HETE and 12-HETE in UC correlated with calprotectin. This confirms the fact that UC is more strongly associated with the HETE synthesis pathway and calprotectin concentration. Monitoring their concentrations would prevent a more significant infiltration of neutrophils into intestinal tissues and, consequently, the release of the acute-phase protein calprotectin. This provides an opportunity for the early intervention and inhibition of the active phase in the early stage. Thanks to this, patients could stay at home during outpatient treatment, which would improve their comfort and quality of life while also reducing costs.

The limitation of this study is its sample size, which, even though patient data were derived from two different centers, is not very large. The children were of Caucasian ethnicity, which constitutes approximately 98% of the population. Additionally, due to the ages of the subjects (14/04 + 2.93), parents and legal guardians did not consent to blood collection from healthy children. Therefore a control group could not be established, and we could only compare results within the diseased subjects, which is also a limitation of this study. 

## 5. Conclusions

- Calprotectin showed a positive correlation with 12- and 5-HETE acids in UC. This implies that it is associated with the synthesis of HETE acids and indicates the possible involvement of the 5- and 12-HETE synthesis pathways in the activation of neutrophils and the release of calprotectin in UC.
- The activity of UC itself is associated with the activation of the 16 RS HETE pathway.
- UC does not show a relationship with HODE acids. The duration of UC intensifies the synthesis of the 15 HETE and 5 oxo ETE pathways. This dependence does not apply to CD, nor to all derivatives of HETE acids, in which no correlation with calprotectin was detected. Calprotectin could possibly serve as an early diagnostic element in monitoring UC. Patients with CD exhibit higher concentrations of KT metabolites, suggesting a more severe course of CD compared to UC. In CD, the concentration of 12S HETE also decreases with age. Both diseases should be considered separately due to the differences in their individual profiles.
- In order to ensure the reliability of the results of this study, tests should be performed on a larger group, taking into account differences in age and ethnic origin.

## Figures and Tables

**Table 1:** Characteristic of the study group.

Parameter	CD and UC		CD		UC		*p*-Value
	Avg.	SD	Avg.	SD	Avg.	SD	
Age (years)	14.036	2.928	13.752	2.733	14.359	3.152	0.209
Body weight (kg)	50.132	18.907	46.830	18.351	53.890	19.142	0.143
Height (cm)	1.572	0.194	1.528	0.192	1.611	0.192	0.139
BMI (kg/m^2^)	19.608	4.633	19.142	4.332	20.139	4.977	0.401
BMI percentile	44.717	36.194	44.161	35.781	45.310	37.185	0.681
Body weight percentile	42.140	35.933	39.884	35.433	44.552	36.748	0.443
Onset of disease (months)	11.800	3.933	11.205	3.420	12.478	3.757	0.166
Disease duration (months)	24.355	31.174	28.424	31.450	19.724	30.742	0.2765
Calprotectin (µg/g)	2053.032	2181.252	1978.952	2236.876	2166.620	2165.336	0.799
Disease activity index PCDAI or PUCAI	22.834	20.971	16.214	16.234	30.833	23.483	0.010

Avg.—average; SD—standard deviation; PCDAI—pediatric Crohn’s disease activity index; PUCAI—pediatric ulcerative colitis activity index.

**Table 2:** Characteristics of lipid mediators in CD and UC.

Metabolite	UC and CD		UC		CD		*p*-Value
	Avg.	SD	Avg.	SD	Avg.	SD	
PGE2	9.716	28.462	8.987	27.123	10.357	29.994	0.851
LTX A4 5S. 6R	0.101	0.124	0.103	0.133	0.100	0.118	0.911
LTX A4 5S. 15R	0.089	0.102	0.084	0.086	0.093	0.115	0.732
Leukotriene B4	0.104	0.083	0.099	0.073	0.108	0.092	0.664
16RS HETE	0.577	0.494	0.621	0.399	0.540	0.568	0.524
13S HODE	0.339	0.380	0.281	0.295	0.390	0.440	0.263
9S HODE	0.398	0.424	0.329	0.320	0.459	0.494	0.233
15S HETE	0.958	0.665	0.811	0.413	1.087	0.810	0.103
12S HETE	3.443	3.110	3.491	3.783	3.400	2.430	0.909
5 oxo ETE	0.930	0.849	0.965	0.913	0.900	0.803	0.768
5 HETE	2.227	1.622	2.077	1.876	2.360	1.378	0.497
TXB2	0.086	0.074	0.081	0.063	0.91	0.083	0.575

PGE2—prostaglandin E2; LTX—lipoxins; LTB4—leukotriene B4; HETE—hydroxyeicosatetraenoic acid; HODE—hydroxyoctadecadienoic acid; TXB2—tromboxan 2; oxo ETE-metabolite; CD—Crohn’s disease; UC—ulcerative colitis; Avg.—average; SD—standard deviation, *p*-value < 0.05.

**Table 3:** Mean contents of analyzed fatty acid metabolites, considering type and disease activity index.

Metabolite	UC and CD				UC				CD			
	Avg.	SD	PCDAI/PUCAI	*p*-Value	Avg.	SD	PUCAI	*p*-Value	Avg.	SD	PCDAI	*p*-Value
PGE2	9.716	28.46	−0.003	0.400	8.987	27.12	0.191	0.082	10.357	29.99	−0.188	0.041 *
LTX A4 5S. 6R	0.101	0.124	−0.263	0.032 *	0.103	0.133	−0.346	0.238	0.100	0.118	−0.227	0.696
LTX A4 5S. 15R	0.089	0.102	−0.301	0.092	0.084	0.086	−0.401	0.103	0.093	0.115	−0.302	0.215
Leukotriene B4	0.104	0.083	0.217	0.736	0.099	0.073	0.368	0.118	0.108	0.092	−0.008	0.489
16RS HETE	0.577	0.494	0.154	0.073	0.621	0.399	0.716 *	0.235	0.540	0.568	−0.294	0.302
13S HODE	0.339	0.380	−0.088	0.259	0.281	0.295	0.246	0.620	0.390	0.440	−0.346	0.524
9S HODE	0.398	0.424	−0.099	0.379	0.329	0.320	0.175	0.803	0.459	0.494	−0.249	0.375
15S HETE	0.958	0.665	−0.251	0.071	0.811	0.413	−0.144	0.105	1.087	0.810	−0.270	0.075
12S HETE	3.443	3.110	0.047	0.878	3.491	3.783	0.174	0.279	3.400	2.430	−0.377	0.005 *
5 oxo ETE	0.930	0.849	−0.125	0.124	0.965	0.913	−0.003	0.377	0.900	0.803	−0.457 *	0.336
5 HETE	2.227	1.622	0.051	0.614	2.077	1.876	0.221	0.569	2.360	1.378	−0.222	0.163
TXB2	0.086	0.074	0.040	0.565	0.081	0.063	0.453	0.744	0.91	0.083	−0.182	0.217

PGE2—prostaglandin E2; LTX—lipoxins; LTB4—leukotriene B4; HETE—hydroxyeicosatetraenoic acid; HODE—hydroxyoctadecadienoic acid; TXB2—tromboxan 2; CD—Crohn’s disease; UC—ulcerative colitis; Avg.—average; SD—standard deviation, *p*-value < 0.05. *—statistically significant difference; PCDAI-Pediatric Crohn’s Disease Activity Index; PUCAI- Pediatric Ulcerative Colitis Activity Index, oxo ETE-metabolite.

**Table 4:** Correlations of proinflammatory mediators with calprotectin and disease index in the overall IBD group, as well as UC and CD.

All Groups	TXB2	PGE2	LTX A4 5S. 6R	LTX A4 5S. 15R	Leucotrien B4	16 RS HETE	13S HODE	9S HODE	15S HETE	12S HETE	5 oxo ETE	5 HETE
Calprotectin IBD	0.171	−0.112	0.066	−0.033	0.314	0.153	−0.053	−0.020	−0.223	0.270	−0.099	0.212
DAI-IBD	0.040	−0.003	−0.269	−0.301	0.217	0.154	−0.088	−0.099	−0.251	0.047	−0.125	0.051
Calprotectin UC	0.308	0.083	0.407	−0.005	0.387	0.479	0.074	0.102	−0.190	0.653 *	−0.093	0.668 *
DAI-UC	0.453	0.191	−0.346	−0.401	0.368	0.716 *	0.246	0.175	−0.144	0.174	−0.003	0.221
Calprotectin CD	0.117	−0.221	−0.070	−0.049	0.262	0.009	−0.120	−0.072	−0.244	−0.163	−0.119	−0.264
DAI-CD	−0.182	−0.188	−0.227	−0.302	−0.008	−0.294	−0.346	−0.249	−0.270	−0.377	−0.457 *	−0.222

PGE2—prostaglandin E2; LTX—lipoxins; LTB4—leukotriene B4; HETE—hydroxyeicosatetraenoic acid; HODE—hydroxyoctadecadienoic acid; TXB2—tromboxan2; oxo ETE-metabolite;DAI—disease activity index; IBD—inflammatory bowel diseases; CD—Crohn’s disease; UC—ulcerative colitis; deviation, *p*-value < 0.05. * statistically significant difference.

## Data Availability

Data are available upon request.
